# A Case of Adenocarcinoma of Uterus Masculinus in a Pomeranian Dog

**DOI:** 10.3389/fvets.2020.00337

**Published:** 2020-06-16

**Authors:** Massimo Vignoli, Ippolito De Amicis, Roberto Tamburro, Gina Quaglione, Nicoletta Salviato, Francesco Collivignarelli, Rossella Terragni, Stefano Pastrolin, Giuseppe Marruchella

**Affiliations:** ^1^Faculty of Veterinary Medicine, University of Teramo, Teramo, Italy; ^2^Human Pathology Unit, “Giuseppe Mazzini” Hospital, Piazza Italia, Teramo, Italy; ^3^Poliambultorio Cà Sillis, Venezia, Italy; ^4^Pet Care Veterinary Clinic, Bologna, Italy; ^5^San Francesco Veterinary Hospital Diagnostic, Treviso, Italy

**Keywords:** uterus masculinus, Müllerian duct, adenocarcinoma, dog, computed tomography

## Abstract

**Introduction:** Persistent Müllerian duct syndrome (PMDS), or uterus masculinus, is a rare autosomal recessive form of male pseudohermaphroditism due to the failure of paracrine anti-Müllerian hormone (AMH) secretion by Sertoli cells or failure of the Müllerian ducts to respond to AMH secretion. The malignant degeneration of persistent Müllerian remnants is rare. In human medicine, few related reports exist. In veterinary medicine, this is the first report describing adenocarcinoma of the uterus masculinus involving the prostate in a dog.

**Clinical history:** An 11-year-old, male, neutered Pomeranian dog was referred for computed tomography due to the suspicion of prostatic carcinoma based on ultrasound and cytological examinations. The computed tomography findings were consistent with a uterus masculinus mass with possible prostatic infiltration. Uterus masculinus removal and total prostatectomy were performed; termino-terminal urethral anastomosis was carried out. Dehiscence of the anastomosis was observed 3 days after surgery. The owner declined any further procedures, and the dog was euthanized 5 days after surgery. Histopathological evaluation revealed adenocarcinoma of the uterus masculinus.

**Conclusion:** Adenocarcinoma of the uterus masculinus may occur, suggesting that patients with PMDS should be evaluated for malignant changes of Müllerian remnants.

## Background

Persistent Müllerian duct syndrome (PMDS), or uterus masculinus, is a rare condition in both male humans and dogs, in which the embryological remnant of the Müllerian duct system has not regressed (1, 2).

The Müllerian duct system is present in both male and female mammalian embryos (3, 4). In normal females, it differentiates into the oviducts, uterus, cervix, and cranial portion of the vagina, while in normal males, the Müllerian duct system regresses soon after testicular development (3, 4).

Müllerian inhibiting substance (MIS) also called anti-Mullerian hormona (AMH) is secreted by Sertoli cells and is responsible for regression of Mullerian ducts during embryonic development in male mammals. However, Mullerian structures (uterus, oviducts, cervix and cranial vaginal) could be present in dogs with Müllerian duct syndrome (PMDS) (5).

In canine PMDS, the affected dog appears masculine in all outward appearances, but can retain their Müllerian structures. However, PMDS should be suspected in cryptorchid males, mainly in miniature Schnauzers (5).

MDS is known to be an inherited autosomal recessive disorder in miniature Schnauzers (1). It has been hypothesized that an absent or defective AMH receptor (AMHR2) and not a failure of Sertoli cells to secrete AMH or AMH inactivity is responsible for the presence of Müllerian structures in males (6).

PMDS or uterus masculinus is a rare condition in dogs. Sometimes it is a coincidental finding during abdominal diagnostic imaging or surgery. However, it may also be detected because of associated disorders, such as urinary tract infection, pyometra, hydrometra, cystic uterus, prostatitis, cryptorchidism or testicular neoplasia (5). Recently, the first case of a uterine leiomyoma in a miniature Schnauzer dog was reported (7).

A primary tumor involving the Müllerian duct remnants has never been reported in veterinary literature.

Thus, the aim of this report was to describe a unique case of adenocarcinoma of uterus masculinus in a Pomeranian dog.

## Case Presentation

An eleven-year-old, neutered, male Pomeranian dog with a body weight of 2.7 kg was referred for computed tomography (CT) due to the suspicion of prostatic carcinoma after the ultrasound (US)-guided cytological examination performed by the referring veterinary surgeon. On anamnesis, the dog was neutered at 1 year of age. The owner reported observing frequent and difficult urination and blood in the urine over the last 2 months. Clinically, the dog presented with pain on digital rectal prostatic examination and an irregular dorsal prostate surface.

Biochemistry and hematology analyses did not show any relevant features. Urinalysis showed an erythrocyte level of 250/μl (> 50 HPF), a leucocyte level of 100/μl (> 50 HPF), and an epithelial non-squamous cell level of 3–5/HPF. The urine culture result was negative.

**Figure 1:**
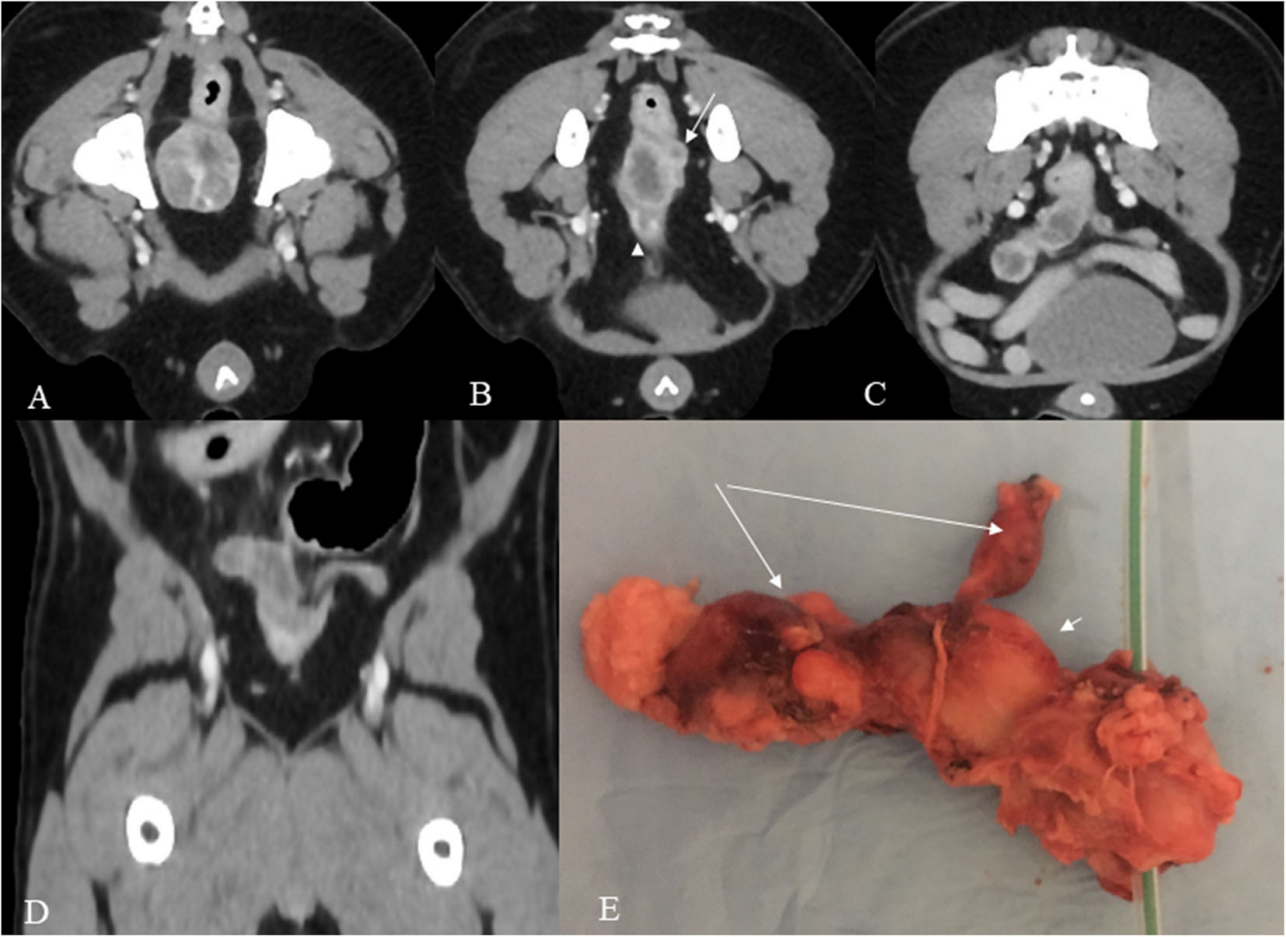
**(A–E)** Post contrast venous phase CT images **(A–D)** and gross anatomy **(E)**. The prostate shows heterogeneous enhancement in this mid-prostate cross section image **(A)**. In the cross sectional image dorsocranial to the prostate **(B)**, a hypoattenuating oval tubular structure with peripheral contrast enhancement is present consistent with the body of the uterus and a second smaller tubular structure dorsally and to the left consistent with the left horn (arrow). A urethra is also shown in the figure (arrowhead). More cranially, both uterine horns are visible, the right with severely increased volume and both with a wavy appearance **(C)**. The dorsal reconstruction shows both horns **(D)**. The specimen **(E)** shows the prostate with a urethral catheter, the body of the uterus (short arrow), and the two uterine horns (long arrows).

Pre and post intravenous contrast medium administration whole body CT showed a heterogeneous attenuation prostate gland measuring 2,4 mm in diameter with focal mineralization in the left lobe (Figures 1A–D).

Dorsal to the prostate, an irregular tubular structure with no cleavage plane with the prostate was present. It continued cranially, ending with two asymmetrical horns.

The dog underwent surgery 2 weeks later. No medication was administered in the interim. Before surgery, US was performed and showed that the body of the uterus masculinus and the left horn were heterogeneous, while the right horn was mainly hypoechoic. All structures showed wavy but well-defined borders. The maximum thickness of the uterus masculinus was 16 mm in the body, 13 mm in the right horn, and 10 mm in the left horn. The total length was approximately 22 mm for the body, 30 mm for the right horn, and 14 mm for the left horn.

### Anesthesia Protocol

The dog was sedated with morphine (0.2 mg/kg, IM) and acepromazine (0.05 mg/kg, IM) 20 min before induction. After 5 min of preoxygenation, anesthesia was induced with propofol (4 mg/kg, IV). Cefazoline (20 mg/kg, IV) was administered at induction. The patient was then intubated with a cuffed endotracheal tube, and a stable level of anesthesia was achieved with sevoflurane and oxygen with the patient positioned in dorsal recumbency.

### Surgical Procedure

A midline celiotomy from the xiphoid process to the pubic bone was performed. Surgical exploration of the caudal abdomen revealed the presence of a uterus with two small horns and evidence of prostatomegaly. The uterine body with the two cranially oriented horns was located and attached dorsally to the prostate.

The surgical procedure aimed to remove the mass involving the uterus masculinus in association with total prostatectomy.

To increase exposure of the urethra, bilateral pubic and ischial osteotomy was carried out. The division between the right and left adductor muscles was incised sharply, taking care to stay exactly on the midline to minimize hemorrhage. The adductor muscles were then elevated subperiosteally from the pubis and ischium with a one-fourth inch Keys periosteal elevator. Elevation was continued until the obturator nerves and approximately two-thirds of the obturator foramina were visible. The prepubic tendon was incised along the pubis to the level of the proposed pubic osteotomy sites. A 2 mm pin chuck was used to drill 2 holes on either side of the 4 proposed osteotomy sites. The right and left pubis and ischium were then osteotomized with a sagittal oscillating saw. The internal obturator nerves were protected with a malleable retractor while osteotomy was performed. The internal obturator muscle was elevated subperiosteally from the right pubis and ischium, allowing resection of the central bony plate to the left, and the idiopathic prostatic gland was identified through palpation. The urethral sagittal incision was then performed caudal to the prostate. Stay sutures were placed to prevent rotation of the urethral stump, a Foley catheter was advanced across the defect into the bladder, and the retaining balloon was inflated. End-to-end anastomosis with simple interrupted sutures using a 4-0 monofilament absorbable suture material was performed. The bony plate was reduced, 2-0 polydioxanone sutures were placed through the previously drilled holes at each osteotomy site and secured. The adductor muscles were reapposed with 3-0 polydioxanone sutures in a cruciate pattern. The prepubic tendon was secured to the pubis with 3-0 polydioxanone sutures in a simple interrupted pattern. The linea alba was closed with 3-0 polydioxanone sutures in a simple continuous pattern, and subcutaneous tissues and skin were closed routinely.

On gross inspection, the excised mass clearly resembled a uterus, consisting of a main central body and two asymmetrical “horns.” The mass was quite firm on palpation; on cross-sectional observation, it appeared homogeneously whitish-gray, with no lumen evident (Figure 1E).

The entire excised mass was fixed in 10% buffered formalin; then, representative samples were collected from the body and both horns, embedded in paraffin and routinely processed for histopathological (hematoxylin and eosin staining) and immunohistochemical investigations. *Postoperative management*

The dog recovered under close supervision in the intensive care unit. Postoperative medication included methadone (0.2 mg/kg, IM, every 4–6 h), intravenous cefazolin (22 mg/kg, every 8 h), and continuous intravenous 0.9% NaCl crystalloid therapy. Postoperative complications were classified as major in the event of surgical revision and as minor in the case revisional surgery was not required.

Three days later, dehiscence at the urethral sutures with abdominal effusion was observed as a complication. Surgical revision was suggested, but worsening of the patient's condition led the owner to choose euthanasia.

**Figure 2:**
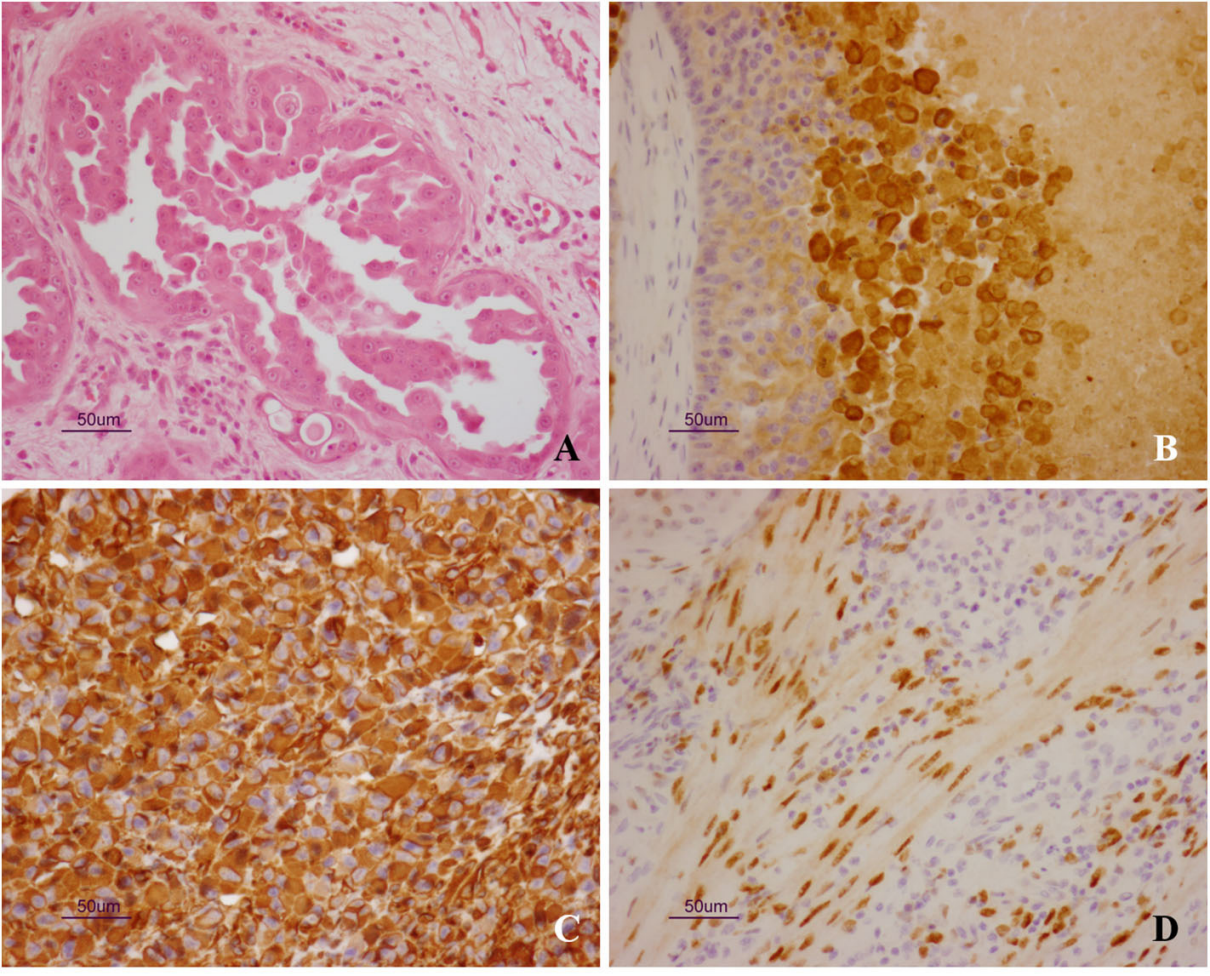
**(A–D)** Uterus masculinus. Histopathological and immunohistochemical findings. **(A)** Neoplastic cells arranged in micropapillary projections. Scattered, mononuclear inflammatory cells are seen within the surrounding stroma (hematoxylin and eosin stain). Strong and specific immunoreactivity for cytokeratin-7 **(B)** and vimentin **(C)** is demonstrated inside the cytoplasm of neoplastic cells. Immunolabeling for estrogen receptor α is also observed inside the nuclei of smooth muscle cells **(D)**.

Microscopically, epithelial neoplastic proliferation was observed, diffusely infiltrating the entire mass. Neoplastic cells were large and polygonal-shaped, with a single nucleus and abundant cytoplasm. Nucleoli were prominent, and mitotic figures were also commonly observed (0-2/HPF). The arrangement of neoplastic cells varied throughout the mass, from solid to cystic-papillary in appearance (Figure 2A). Large areas of necrosis were also observed, along with the deposition of cholesterol crystals. Inflammatory infiltrates were noted at the edge of the neoplasm, while foci of purulent inflammation occurred inside necrotic foci. On immunohistochemistry, neoplastic cells were positive for cytokeratin-7 (Dako Omnis, clone OV-TL 12/30; Figure 2B) and vimentin (Dako Omnis, clone V9; Figure 2C); moreover, smooth muscle cells and occasional neoplastic cells showed nuclear immunoreactivity for estrogen receptor α (Dako Omnis, clone EP1; Figure 2D). The neoplastic process infiltrated the contiguous prostate, showing the same microscopic features.

Based on the histopathological findings, the diagnosis of persistent Müllerian duct adenocarcinoma was made.

## Discussion

In humans, uterus masculinus has been defined as the persistence and subsequent formation of female genital organs (uterus, fallopian tubes, upper vagina) in normal males (2). Adenocarcinoma of the uterus masculinus has never been diagnosed in dogs; in human medicine, a few related cancers have been reported (2, 8–10).

In 2002, Shinmuta et al. (9) reported a case of adenocarcinoma of the uterus masculinus as an incidental finding discovered during the autopsy of a 67-year-old patient who died from a road traffic accident. A case of uterine adenosarcoma was first described in a 14-year-old boy by Thiel and Herald in 2005 (8). Dimasis et al. (2) reported a case of high-grade uterine adenocarcinoma in a 45-year-old man with a 3-month history of painless hematuria and abdominal protuberance. Both of these patients underwent Müllerian remnant resection by a transabdominal approach; the surgeons reported very difficult dissection of the mass from the surrounding tissue (2). No surgical complications were reported, but the patients developed severe metastatic invasion 2 and 3 months after surgery (2). Kovachev et al. (10) reported a case of uterine leiomyoma in a 46-year-old man in 2014. Abdominal orchiectomy and hysterectomy were performed. The postoperative course was uneventful, and the patient subsequently underwent radiation and chemotherapy. At the last follow-up, the patient was alive with no evidence of disease (10).

In the veterinary medicine literature, a primary tumor of the uterus masculinus has never been reported (1, 7, 11–16).

In 1986, Atilola et al. (16) described six dogs affected by cystic uterus masculinus. One patient had transitional cell carcinoma of the prostate, and another dog developed a Sertoli cell tumor of the right testicle.

In 2009, Matsuu et al. (12) reported the history of a 10-year-old miniature Schnauzer with bilateral cryptorchidism and male external genitalia. This dog was diagnosed with a bilateral Sertoli cell tumor with hydrometra (12). In 2015, Lim et al. (7) described the ultrasonographic findings of six dogs with a uterus masculinus; in one case, lymphomatous malignant infiltration of the remnant female genitalia in the context of multicentric lymphoma was reported. In 2019, Noguera et al. (13) described the case of an 8-year-old miniature Schnauzer (MS) dog with PMDS and bilateral Sertoli cell testicular tumors; the dog died a few days after surgical castration and hysterectomy. In these studies, uterus masculinus was diagnosed using radiography and ultrasonography. On US, the uterus masculinus appeared as single (four dogs) or two (two dogs) horn-like, tubular (four dogs) or cylindrical (two dogs) structures, originating from the craniodorsal aspect of the prostate gland and extending cranially. The walls of the uterus masculinus were isoechoic relative to the urinary bladder wall (11, 16).

The management of PMDS may be conservative (2, 8). In cases of severe clinical signs, surgical excision of the Müllerian remnants has been the recommended treatment. Most dogs do well-postoperatively, although prostatectomy has never been necessary (11, 16).

In the present report, we presented a unique case of adenocarcinoma of the uterus masculinus involving the prostate in a neutered Pomeranian dog. The patient was referred with a diagnosis of suspected prostatic adenocarcinoma obtained by fine-needle aspiration (FNA) performed under US guidance. Tumor staging achieved by CT allowed us to exclude any metastatic lesions and to observe the presence of a mass dorsocranially to the prostate consistent with uterus masculinus.

Enhanced CT of the pelvis showed a heterogeneous enhancement of the prostatic gland and dorsocranial to the prostate a well-defined, tubular, with thick wall structure consistent with the uterus that showed peripheral contrast enhancement and extending to the right inguinal area. In reconstruction images both uterine horns were evident. The above CT findings were of paramount importance in the diagnosis, leading to the uterus masculinus mass.

CT is an excellent imaging modality for evaluating the abdomen and unlike US is not limited by intestinal gas. Moreover, it can be used to confirm ultrasonographic findings and allows a more thorough presurgical evaluation of the abnormal anatomy (5). In human medicine, CT and magnetic resonance imaging (MRI) are established imaging modalities for the diagnosis and follow-up of Müllerian anomalies (2, 6, 8, 17, 18). In humans, MRI is the modality of choice for examining primary tumors, while CT has been used to check for metastasis (2, 8). Although MRI is considered superior to CT in evaluating soft tissue structures (18), abdominal MRI is not commonly performed in veterinary medicine due to reduced availability, especially in the case of high-field-strength units. Surgery was performed to remove the tumor involving the persistent Müllerian duct remnants. At the time of surgery, no evidence of metastatic lesions was observed. Because of the neoplastic involvement of the prostate, the surgical procedure included en bloc Müllerian remnant removal in association with total prostatectomy (19). As reported in the literature, the uterus masculinus arose from the craniodorsal portion of the prostate and extended cranially. Such a close anatomical connection reasonably justifies the neoplastic infiltration of the prostate (11).

Complications associated with total prostatectomy include urethra incisional dehiscence, urinary incontinence, hemorrhage and uroabdomen (19). Due to complications and worsening of the overall clinical condition in this case, the dog was euthanized 5 days after surgery.

The main limitation of this study is related to the lack of information regarding the history of the dog during the first year of life. In particular, as the dog was neutered at 1 year of age, we were not able to detect any further pathology that may be associated with PMDS.

In conclusion, adenocarcinoma of the uterus masculinus may occur, suggesting that patients with PMDS should to be evaluated for malignant changes of Müllerian remnants.

## Data Availability Statement

All datasets presented in this study are included in the article/supplementary files.

## Ethics Statement

Ethical review and approval was not required for the animal study because the study was a case report, thus no ethical approval was required. Written informed consent was obtained from the owners for the participation of their animals in this study. Written informed consent was not obtained from the individual(s) for the publication of any potentially identifiable images or data included in this article.

## Author Contributions

MV, ID, RTa, GQ, and GM wrote the paper. MV, RTe, NS, and SP were involved in the Imaging assessment. FC, RTa, and MV performed the surgical procedure. GM was in charge for the histopathological evaluation. MV was responsible for the final content. All of the authors have read and approved the final manuscript.

## Conflict of Interest

The authors declare that the research was conducted in the absence of any commercial or financial relationships that could be construed as a potential conflict of interest.
